# Supplementary material for: Protocol for the evaluation of cost-effectiveness and health equity impact of a school-based tobacco prevention programme in a cluster randomised controlled trial (the TOPAS study)
Source: BMJ Open. 2021 Aug 11;11(8):e045476. doi: 10.1136/bmjopen-2020-045476 (PMC8362718; doi:10.1136/bmjopen-2020-045476)
Supplement: Author's manuscript [file bmjopen-2020-045476.draft_revisions.pdf]

BMJ Open

BMJ Open is committed to open peer review. As part of this commitment we make the peer review history of every article we publish publicly available.

When an article is published we post the peer reviewers' comments and the authors' responses online. We also post the versions of the paper that were used during peer review. These are the versions that the peer review comments apply to.

The versions of the paper that follow are the versions that were submitted during the peer review process. They are not the versions of record or the final published versions. They should not be cited or distributed as the published version of this manuscript.

BMJ Open is an open access journal and the full, final, typeset and author-corrected version of record of the manuscript is available on our site with no access controls, subscription charges or pay-per-view fees (http://bmjopen.bmj.com).

If you have any questions on BMJ Open's open peer review process please email info.bmjopen@bmj.com

BMJ Open

Protocol for the evaluation of the cost-effectiveness and health equity impact of a school-based tobacco prevention programme in a cluster randomised controlled trial (The TOPAS Study)

Journal:	*BMJ Open*
Manuscript ID	bmjopen-2020-045476
Article Type:	Protocol
Date Submitted by the Author:	03-Oct-2020

Complete List of Authors:	Pulkki-Brännström, Anni-Maria; Umea University, Department of Epidemiology and Global Health<br>Galanti, Maria; Karolinska Institutet, Department of Global Public Health<br>Nilsson, Maria; Umea University, Department of Epidemiology and Global Health

Keywords:	HEALTH ECONOMICS, PUBLIC HEALTH, PREVENTIVE MEDICINE

SCHOLARONE™
Manuscripts

I, the Submitting Author has the right to grant and does grant on behalf of all authors of the Work (as defined in the below author licence), an exclusive licence and/or a non-exclusive licence for contributions from authors who are: i) UK Crown employees; ii) where BMJ has agreed a CC-BY licence shall apply, and/or iii) in accordance with the terms applicable for US Federal Government officers or employees acting as part of their official duties; on a worldwide, perpetual, irrevocable, royalty-free basis to BMJ Publishing Group Ltd ("BMJ") its licensees and where the relevant Journal is co-owned by BMJ to the co-owners of the Journal, to publish the Work in this journal and any other BMJ products and to exploit all rights, as set out in our licence.

The Submitting Author accepts and understands that any supply made under these terms is made by BMJ to the Submitting Author unless you are acting as an employee on behalf of your employer or a postgraduate student of an affiliated institution which is paying any applicable article publishing charge ("APC") for Open Access articles. Where the Submitting Author wishes to make the Work available on an Open Access basis (and intends to pay the relevant APC), the terms of reuse of such Open Access shall be governed by a Creative Commons licence – details of these licences and which Creative Commons licence will apply to this Work are set out in our licence referred to above.

Other than as permitted in any relevant BMJ Author's Self Archiving Policies, I confirm this Work has not been accepted for publication elsewhere, is not being considered for publication elsewhere and does not duplicate material already published. I confirm all authors consent to publication of this Work and authorise the granting of this licence.

For peer review only

Protocol for the evaluation of the cost-effectiveness and health equity impact of a school-based tobacco prevention programme in a cluster randomised controlled trial (The TOPAS Study)

Anni-Maria Pulkki-Brännström*[1], Maria Rosaria Galanti[2] and Maria Nilsson[1]

[1] Department of Epidemiology and Global Health, Umeå University, Umeå, Sweden

[2] Department of Public Health Sciences, Karolinska Institutet, Stockholm, Sweden

* Corresponding author: Anni-Maria Pulkki-Brännström, Department of Epidemiology and Global Health, Umeå University, Sweden. anni-maria.pulkki-brannstrom@umu.se

Word count: 3787

For peer review only

ABSTRACT

Introduction

Despite a long-term downward trend in smoking prevalence, tobacco remains the number one risk factor for death and disability in Sweden. Globally, tobacco use generates a substantial economic burden for health systems and is also a major driver of socioeconomic inequalities in health. This paper describes the planned evaluation of the cost-effectiveness and health equity impact of a multi-component school-based programme to prevent the onset of tobacco use in adolescents.

Methods and analysis

The TOPAS study combines a two-arm cluster randomised controlled trial and an observational study. Cost-effectiveness of the multi-component Tobacco-Free Duo programme will be evaluated against the educational component of the same programme only. Incremental cost-effectiveness ratios (ICERs) will be calculated in termsof i) the cost per case prevented – using the trial primary outcome and within-trial payer costs; and ii) cost per life-year saved – using all trial outcomes and costs from a limited societal perspective. If ICERs are negative, incremental net benefit ratios will be calculated. Robustness of the results will be assessed through one-way sensitivity analyses. The slope index of inequality will be computed to assess the potential impact of the Tobacco-free Duo programme on i) socioeconomic inequalities in the onset of tobacco use and adult smoking cessation, comparing the two trial arms, and ii) differences in the prevalence of adolescent ever-smokers between schools with different socioeconomic profiles, comparing time trends with external reference schools, which represent usual practice in the observational study.

Ethics and dissemination

Ethical approval was obtained from the regional ethics committee in Umeå (Registration number 2017/255-31). The Public Health Agency of Sweden commissioned the study. The findings will be disseminated internationally within academia and to national and local policy-makers.

Trial registration number ISRCTN 52858080; Pre-results.

For peer review only

STRENGTHS AND LIMITATIONS OF THIS STUDY

- This study uses primary data and an experimental design to address the lack of rigorous economic evaluations of tobacco-prevention programmes.
- The results will mirror the real costs and cost-effectiveness of the programme, because the intervention is implemented by regular school personnel and integrated into schools' routine tobacco prevention activities.
- The study includes a health equity impact analysis in which the full programme (T-Duo) is compared with structured classroom education only (E) and also with current practice, represented by reference schools.
- Intervention effects are measured on the individual level but resource use is measured on the level of the school.
- The study will not be able to capture the support that the adult "pair" provides to the adolescent in keeping up the "tobacco-free agreement".

For peer review only

For peer review only - http://bmjopen.bmj.com/site/about/guidelines.xhtml

BACKGROUND

Tobacco use is a persistent global public health challenge, and scientific evidence of the effects of tobacco use on mortality and morbidity across the life course is robust. Around 50% of adult smokers die prematurely from tobacco-related disease (1). Smoking is the single most preventable cause of death and one of the leading causes of inequity in health (2). Furthermore, smoking poses large economic costs to individuals, health care systems and the society at large. The World Health Organization has estimated that the economic harm caused by smoking to the world economy is over US$ 500 billion each year (3). The benefits from reduced tobacco use are undisputable, and encompass improved population health, reduced health inequalities, reduced health care costs, and improved economic productivity.

The young person becomes a smoker in a social context (4). Many programmes to prevent young people's tobacco use employ methods based on social influence models. The essence of such models is to change the attitudes, knowledge and behaviour of young people in the context of a social environment. Parents, siblings and friends exert social influence, and the more smokers a young person has in the close context, the more will start smoking (5). Individual and contextual factors influence the process from initiation to regular smoking, and the individual risk of becoming a smoker is impacted by how the different factors relate to and interact with each other.

The need for evidence-based tobacco-prevention programmes targeting young people is large. Programmes implemented outside the health care sector, such as in schools, potentially reach all adolescents, and align well with global calls for multisectoral approaches to promoting health and wellbeing. However, relatively few rigorous evaluations exist. The Swedish agency responsible for recommendations in this area reviewed the literature and found the evidence base to be insufficient, and many evaluation methodologies to be inappropriate, to support recommendations for interventions targeting young people (6). A recent systematic review specifically of the health economic evidence on school-based interventions by Leão et al. (7) found that few evaluations had been published, particularly not recently. Therefore, more evidence is called for.

For peer review only - http://bmjopen.bmj.com/site/about/guidelines.xhtml

For new evidence to be relevant for policy-makers, researchers should analyse cost-effectiveness and budget impact, and effects on health inequalities as well as on average health outcomes (8). The large socioeconomic inequalities in smoking are well-documented, and parents' low socioeconomic status is a risk factor for adolescent smoking initiation (9). However, a number of studies highlight that inequalities among adolescents are smaller than among adults (10). To our knowledge, no evaluation has as yet focused on whether smoking prevention among adolescents has any effects on the socioeconomic inequalities in smoking.

TOPAS is a three-year intervention study that evaluates a multi-component school-based programme with public commitment to prevent the onset of tobacco use in adolescents in Sweden. The study employs a mixed design approach and consists of a two-arm cluster randomised controlled trial (cRCT) and an observational study. In the cRCT, thirty-four lower secondary schools (clusters) are randomised to either the comprehensive Tobacco-free Duo programme or structured classroom education only. The Tobacco-free Duo programme consists of six components: 1) the tobacco-free pair; 2) information about the programme to students; 3) information about the programme to guardians; 4) a membership card for adolescents who form a tobacco-free pair; 5) yearly disclosure by the tobacco-free pair of their tobacco-free status; and 6) structured classroom education. The tobacco-free pair is created when the adolescent and a significant adult (e.g. parent) sign a contract by which each party promises to refrain from using tobacco until the adolescent finishes compulsory school (9th grade). In the 17 schools randomised to the comprehensive programme, all adolescents who started 7th grade in autumn 2018 are exposed to all six components for three school years. In the 17 schools randomised to the education-component only, adolescents are exposed only to the sixth component. The observational study includes an additional 16 schools that continue with usual tobacco prevention and health promotion practice. Details of the TOPAS study and the mixed design effectiveness evaluation are described in Galanti et al. (11). This protocol paper aims to fully describe the methodology for the evaluation of the cost-effectiveness and health equity impact of the Tobacco-free Duo programme.

For peer review only - http://bmjopen.bmj.com/site/about/guidelines.xhtml

AIM AND OBJECTIVES

The TOPAS study's health economic evaluation aims to measure the cost-effectiveness and health equity impact of the multi-component Tobacco-free Duo programme. In the cost-effectiveness analysis, the intervention of interest is the full programme (T-Duo) and the comparator is the structured classroom education component of the same programme (E). In the health equity impact analysis, T-Duo is compared with E and also with current practice, represented by reference schools.

The specific objectives are:

1. To conduct a within-trial analysis of the incremental cost-effectiveness of T-Duo against the comparator (E).
2. To estimate the incremental cost-effectiveness of T-Duo taking into account all statistically significant health effects and resource implications beyond the trial period.
3. To assess whether socioeconomic inequalities in the onset of tobacco use and in adult smoking cessation differ between T-Duo, E and current practice.

The current study will be the first to assess the cost-effectiveness of an intervention to prevent adolescent smoking initiation that includes 1) public commitment, and 2) parents or other significant adults (other than school staff). Furthermore, few previous studies have had a well-defined comparator; in our study the comparator is similar to the one in Tengs et al. (12). This study will also be the first to explicitly evaluate the health equity impact of an adolescent smoking prevention programme.

METHODS

Study setting and population

The setting for the TOPAS study is secondary schools in nine regions in central Sweden, ranging from small rural communities to major cities. In Sweden, schools are funded through municipality taxes and run by either municipalities or private providers. Healthcare is funded and organised on a regional level. However, municipalities have some public health responsibilities in the area of tobacco

For peer review only - http://bmjopen.bmj.com/site/about/guidelines.xhtml

prevention, including oversight of national laws and regulations around tobacco, and health promotion in schools.

The main study population consists of adolescents who were in 7th grade in the school year 2018/19 and for whom active consent for data collection has been obtained from a guardian. Secondary study populations are smoking guardians of these adolescents, and all adolescents in 9th grade in three consecutive school years 2018/19, 2019/20, and 2020/21.

Trial design

In the cluster randomised controlled trial, the unit of randomisation (the cluster) is the school. In spring 2018, 34 schools were randomised to either the Tobacco-free Duo programme (T-Duo; 17 schools), or to the structured classroom education component only (E; 17 schools). The intervention started with the training of school staff in August 2018. The implementation period runs until June 2021, corresponding to the three final years of compulsory school for adolescents in the main study population. All data collection will be completed in June 2021.

Detailed information about the school recruitment and randomisation processes, and a full description of the interventions is presented elsewhere (11). A brief description of the six components of the Tobacco-free Duo programme, including the structured classroom education component that is common to both trial arms, and the support that the study team provides to schools for implementation, are presented in the following sections.

The multi-component intervention with public commitment

The overarching aim of the Tobacco-free Duo programme is to prevent tobacco initiation among adolescents using schools as the main arena. The tobacco-free pair (component 1) is a signed agreement between the adolescent and a significant adult. Both parties promise to abstain from tobacco products at least until the adolescent finishes compulsory school. The adolescent is free to choose which significant adult to sign a contract with. Furthermore, the adult is free to choose how best to support the adolescent to keep up the agreement. The programme is introduced by the school

For peer review only - http://bmjopen.bmj.com/site/about/guidelines.xhtml

staff to students (component 2), and to guardians at a regular parents' meeting (component 3). Adolescents who form a tobacco-free pair and sign a contract receive a membership card (component 4). The card is school-specific and each school decides what benefits or discounts cardholders are entitled to; examples include t-shirts and discounts in the school cafeteria. At the end of each school year, all tobacco-free pairs are invited to disclose their tobacco-free status, and adolescents who affirm they have remained tobacco-free enter a prize draw organised by the school (component 5).

Structured classroom education

Structured classroom education is the sixth component of the Tobacco-free Duo programme, and it is common to both trial arms (T-Duo and E). All schools receive lesson materials for two classroom sessions each term. These consist of age-tailored information and practical exercises about tobacco-related topics, e.g. environmental impact. The materials have been externally commissioned for the study.

Support to school staff for implementation

School staff are responsible for implementation of the interventions in their school. School contact persons receive instructions and materials for each programme component from the study team. However, staff retain a degree of independence regarding timing and exact delivery of the intervention components. Support for implementation is given once a year in the form of a training and networking event. The event is organised by the study team but the training is delivered by external, professional trainers with specific expertise in tobacco prevention in schools. Information about topical tobacco-related issues is given, and upcoming lesson materials for the structured classroom education are introduced. The study team also informs about upcoming data collection. To facilitate data collection and retention of schools in the study, the study team is in regular contact with the schools through monthly newsletters, e-mail and telephone.

For peer review only

Measurement of health outcomes

Health outcome data will be collected from adolescents through questionnaires filled in at school, and from guardians through postal questionnaires. Adolescents who change schools will be followed up. Adolescents with missing data on the primary outcome will be excluded from the analysis.

The primary outcome in the cRCT is whether the adolescent has *never smoked* at the end of 9th grade. The primary outcome is measured in summer 2021 among adolescents who attended 7th grade in the intervention schools (T-Duo and E) in autumn 2018, and whose guardians actively consented to data collection. The trial has a number of secondary outcomes including adolescent never smoked regularly; adolescent never used any form of tobacco (e.g. chewing tobacco); and parent has quit smoking (no smoking in the past 30 days). The observational study will provide data on school-level prevalence of adolescent never-smokers for each of the three years covered by the intervention period.

The within-trial cost-effectiveness analysis will use the intention-to-treat results for the trial primary outcome, converted to the number of cases prevented. Thus the average effect of the Tobacco-free Duo programme compared with structured classroom education only will be expressed as the change in the number of adolescents who have *ever smoked* by the end of 9th grade.

To enable comparison with other interventions, a secondary cost-effectiveness analysis will be performed in which the health outcomes will be expressed as life-years. This analysis will aggregate all statistically significant individual-level health effects (whether positive or negative) among both adolescents and parents from the intention-to-treat analyses in the cRCT. For each statistically significant outcome, a literature search will be conducted to obtain an estimate of the associated change in terms of life-years. These estimates will be added up to the total change in life-years, comparing T-Duo with E. For example, if the trial finds a significant reduction in the number of cases prevented (fever ever-smokers by the end of 9th grade), this will be converted to an estimated reduction in the number of adult smokers, and further to expected life-years saved. If sufficient data is available on quality-adjusted life-years, these will be used instead of life-years. Evidence from Sweden will be prioritised. If

For peer review only - http://bmjopen.bmj.com/site/about/guidelines.xhtml

not available, international estimates will be used e.g. from Disease Control Priorities 3 (13) on smoking-related mortality and morbidity.

Measurement of socioeconomic inequalities in the health outcomes

Three health outcome measures will be used in the health equity impact analysis. These are: the onset of tobacco use (trial primary outcome); adult smoking cessation (trial secondary outcome); prevalence of adolescent never-smokers (observational study outcome).

For the individual-level health outcomes, the socioeconomic variable of interest is the highest education level attained by the adolescent's guardian(s). For prevalence, school characteristics will be used as the socioeconomic variables. These include the proportion of students with parents with lower than high school education and the proportion of students with parents born outside Sweden.

Thus, inequalities related to parental education in the onset of tobacco use, and education-related inequalities in adult smoking cessation, will be compared between T-Duo and E; and changes over time in socioeconomic inequalities in school-level prevalence of adolescent never smokers will be compared between T-Duo, E and usual practice (reference schools).

Cost identification, measurement and valuation

Within-trial costs will be collected prospectively from a payer perspective throughout the start-up and implementation phases of the cRCT. These costs consist of resource use i) at the school level, and ii) at the level of the programme.

School-level costs will be collected primarily through an online data collection tool. A link to the survey is sent by email to the school's contact person approximately once a month during term time throughout the trial start-up and implementation periods. The survey contains questions about completed activities, staff time use, and other direct costs for planning and delivery of the intervention. Questions will be included to capture joint costs, such as time used for meetings. Where applicable, data reported by schools will be complemented by records held by the study team. Examples of these

For peer review only

data include attendance lists for the annual training and networking sessions, and the value of lottery prizes for T-Duo component 5. To value staff time use, average national salaries for the reported staff category will be used.

Programme costs will be collected primarily from study financial accounts. These costs include development of intervention materials and the annual training and networking sessions (e.g. staff, refreshments, venue hire, travel). In order not to underestimate total intervention cost, a small proportion of the time used by the principal investigator and the research assistants who have direct contact with schools will be allocated to the intervention. This proportion will be subject to sensitivity analysis. Programme costs will thus cover direct costs and an allocated proportion of study joint costs.

Estimates of the long-term resource consequences associated with each statistically significant trial outcome will be obtained through literature searches. These costs will cover, but not necessarily be limited to, changes in health care costs and in productivity associated with changes in tobacco use. Where available, Swedish estimates from register data or from modelling studies applied to the Swedish setting will be given priority.

All costs will be valued in Swedish kronor (SEK) and inflated to 2021 values using the consumer price index. An annual 3% discount rate will be applied.

Cost-effectiveness and budget impact analyses

Within-trial school- and programme-level start-up and implementation costs for T-Duo and E will be added up respectively. Total costs will be used to estimate unit costs per school, class, and pupil. A within-trial incremental cost-effectiveness ratio will be calculated by dividing the incremental cost with the number of cases prevented, comparing T-Duo with E. If the resulting ratio is negative, incremental net benefit ratios will be calculated instead [14].

The possibility of modelling budget impact and affordability of national scale-up of Tobacco-free Duo will be explored. A municipal payer perspective will be adopted, in

For peer review only - http://bmjopen.bmj.com/site/about/guidelines.xhtml

which any potential healthcare cost savings will be excluded, since healthcare is paid for by regions. For the budget impact analysis, the existence of economies of scale at the level of the school will be analysed through the correlation between observed unit costs and the number of pupils or classes in each school.

To systematically explore uncertainty around cost-effectiveness ratios, one-way sensitivity analyses on key assumptions and programme-level parameters will be conducted. Thus robustness of the within-trial results will be analysed with respect to e.g. staff salary levels, the allocation of joint costs, and the inclusion of start-up costs.

Finally, in a secondary cost-effectiveness analysis, health outcomes will be estimated in terms of life-years, and costs will include estimated changes in healthcare costs and in productivity. Thus this analysis will enable comparison of cost-effectiveness with other interventions, extend the time horizon for health outcomes and costs, and widen the costing perspective from a payer to a partial societal perspective. As far as data available in previous literature allows, a lifetime perspective will be adopted (15).

Equity impact analysis

In line with recent arguments that a focus on relative inequalities is counter-productive given the large need for more evidence of what works to reduce health inequalities (16), this analysis will focus on absolute inequalities. Since the socioeconomic variables are categorical, the slope index of inequality will used for each of the three health outcomes. A descriptive analysis of whether inequalities exist, will first be conducted. The analysis will then examine whether inequalities related to parental education level in the onset of tobacco use at the end of 9th grade differ between T-Duo and E, and whether education-related inequalities in adult smoking cessation differ between T-Duo and E. Third, time trends in the prevalence of ever-smokers at the end of 9th grade between schools with different socioeconomic profiles will be examined, comparing T-duo, E and reference schools. Thus the two first analyses specifically compare the Tobacco-free Duo programme with structured classroom education only, while the third analysis compares these two with each other and with school's usual tobacco prevention practice.

For peer review only - http://bmjopen.bmj.com/site/about/guidelines.xhtml

For peer review only

Patient and public involvement

The intervention under study is a preventive intervention for adolescents and is delivered by regular school staff. School staff are involved in data collection. No patients or members of the public were involved in designing the study.

DISCUSSION

Smoking is the single most preventable cause of death and one of the leading causes of inequity in health. The need for evidence-based tobacco-prevention programmes is large, however few rigorous evaluations exist. There is a particular lack of economic evidence from studies that use recently collected primary data (7). The present study addresses these gaps in the evidence base. Particular attention will be paid to the measurement of programme-level costs beyond the direct costs incurred by the schools, and to the evaluation of health equity impact. The planned study is innovative in that it will provide data to predict system-level costs for this type of intervention (7); and provide evidence to decision-makers on whether the intervention is a good use of public resources for promoting both average health and health equity (8).

Cost-effectiveness analysis was chosen as the evaluation method for a number of reasons. First, cost-effectiveness ratios particularly using quality-adjusted life-years are widely used to inform resource allocation decisions in Swedish health care. Cost-effectiveness is recognised as a priority-setting criteria within the Swedish ethical platform for priority-setting in health care since 1997 (17). Second, unlike many other complex public health interventions that have a variety of health and non-health consequences, the benefits of school-based tobacco prevention are concentrated around the health benefits of reduced tobacco initiation among adolescents. Adolescent smoking status correlates with adult smoking status, which in turn has large implications for mortality, morbidity and healthcare costs. Thus, non-health benefits are of limited concern for the economic analysis, and health benefits are relatively easy to measure in common units even including cessation among smoking

For peer review only - http://bmjopen.bmj.com/site/about/guidelines.xhtml

parents and in a long-term perspective. For these reasons, neither cost-consequence analysis (18) nor the impact inventory method (19) were chosen for this study.

In this study, the within-trial results are considered as a primary analysis, and the estimation of health effects and costs beyond the trial time horizon are treated as secondary. This approach is based on the fact that the societal costs arising from tobacco use are so large that any effective tobacco prevention among adolescents is cost-saving, once the analytical perspective is extended to cover future healthcare cost savings and/or avoided productivity losses (7). Thus, while we accept the need to report on cost-effectiveness using outcome measures that enable comparison with other interventions within and beyond tobacco prevention, we consider that information on the cost per case prevented will be of primary interest to decision-making on whether or not to scale up Tobacco-free Duo within Sweden.

The real-world context for this study is a major strength. The intervention is implemented by regular school personnel and integrated into schools' routine tobacco prevention activities. The study has been designed so that results will mirror, as closely as possible for an experimental setting, the real costs and cost-effectiveness of the programme. Schools will enjoy a high degree of independence regarding how the programme is implemented, which is in line with usual practice in Sweden. Variation in the delivery of the programme components will be captured through the monthly web-based cost collection tool, which includes questions on which intervention components have been delivered in the previous period, to which classes, by whom, and associated time use.

The costs and health effects of implementing a programme in a real-world setting are however uncertain. Socio-demographic characteristics and health behaviours of the participants, as well as the way the intervention is implemented, may differ from the trial setting. Furthermore, cost-effectiveness analysis requires researchers to make a number of assumptions that may substantially affect the resulting cost-effectiveness ratio. We have described how the impact of these assumptions will be systematically assessed. The TOPAS study will provide rich individual- and school-level data that will allow also more extensive analyses. Information from the web-based data collection form will inform the choice of further parameters subjected to sensitivity

For peer review only

analyses. Questions of interest may be how variation in the uptake of the core intervention component – the tobacco-free pair – or schools' implementation of the programme, such as the number of classroom lessons actually delivered, affects the cost-effectiveness ratio.

Common to many other cluster randomised controlled trials of complex public health interventions, individual-level sensitivity analysis is neither possible nor conceptually desirable (20). Intervention effects are measured on the individual level, but the intervention is delivered on the level of the school. Thus exposure and resource use are also measured on the school-level. The only exception is that exposure to the core component, the tobacco-free pair, is measured on the individual level (11). However, the resource use associated with this component – what the adult pair does to support the adolescent in keeping up the agreement – will not be captured in this study.

Dissemination

The results of the study will be disseminated in a scientific article in an international peer-reviewed journal. In addition, results will be disseminated to schools and decision-makers within Sweden through active participation in tobacco prevention – themed events.

Contributorship statement

MRG and MN conceived of and designed the TOPAS study. AMPB and MN conceived the present study. MN acquired the funding. AMPB designed the study, wrote the first draft, and coordinated the write-up of the paper. MN contributed substantially to writing the paper, and together with MRG revised the work critically for important intellectual content. All authors approved of the final version and agree to be accountable for all aspects of the work.

Competing interests

There are no competing interests for any author.

For peer review only - http://bmjopen.bmj.com/site/about/guidelines.xhtml

Funding

The study was funded by the Public Health Agency of Sweden, with case reference number 01346-2017 2.3.2.

Data sharing statement

This is a study protocol; data collection is ongoing and no data are as yet available.

REFERENCES

1. Doll R, Peto R, Boreham J, Sutherland I. Mortality in relation to smoking: 50 years' observations on male British doctors. BMJ. 2004;328(7455):1519.

2. World Health Organization. WHO report on the global tobacco epidemic, 2013: enforcing bans on tobacco advertising, promotion and sponsorship. Geneva, Switzerland: World Health Organization, 2013.

3. World Health Organization. Statement of the World Health Organization in relation to the issue of standardized tobacco product packaging. WHO TRIPS Council Meeting; Geneva 28-29 October 2014. Geneva, Switzerland: World Health Organization, 2014.

4. Lakon CM, Hipp JR, Timberlake DS. The social context of adolescent smoking: a systems perspective. American journal of public health. 2010;100(7):1218-28.

5. U.S. Department of Health and Human Services. Preventing Tobacco Use Among Youth and Young Adults: A Report of the Surgeon General. Atlanta, GA: U.S. Department of Health and Human Services, Centers for Disease Control and Prevention, National Center for Chronic Disease Prevention and Health Promotion, Office on Smoking and Health, 2012.

6. SBU. Att förebygga missbruk av alkohol, droger och spel hos barn och unga (Interventions to prevent misuse of alcohol, drugs and gambling addiction in youth). Stockholm: Swedish Agency for Health Technology Assessment and Assessment of Social Services,, 2015 Contract No.: nr 243.

7. Leão T, Kunst AE, Perelman J. Cost-effectiveness of tobacco control policies and programmes targeting adolescents: a systematic review. European Journal of Public Health. 2018;28(1):39-43.

8. Weatherly H, Drummond M, Claxton K, Cookson R, Ferguson B, Godfrey C, et al. Methods for assessing the cost-effectiveness of public health interventions: Key challenges and recommendations. Health Policy. 2009;93(2-3):85-92.

For peer review only

9. Hedman L, Andersson M, Stridsman C, Ronmark E. Evaluation of a tobacco prevention programme among teenagers in Sweden. BMJ Open. 2015;5:e007673. eng.

10. Vallejo-Torres L, Hale D, Morris S, Viner RM. Income-related inequality in health and health-related behaviour: exploring the equalisation hypothesis. Journal of Epidemiology and Community Health. 2014;68(7):615.

11. Galanti MR, Pulkki-Brännström A-M, Nilsson M. Adult-child contract for universal prevention of tobacco use: Protocol for a mixed-design evaluation of the Swedish school-based program "Tobacco-free Duo" (TOPAS study). JMIR Research Protocols. 2020;(in press). Available at: https://preprints.jmir.org/preprint/21100/accepted.

12. Tengs TO, Osgood ND, Chen LL. The cost-effectiveness of intensive national school-based anti-tobacco education: results from the tobacco policy model. Preventive medicine. 2001;33(6):558-70.

13. Jamison DT, Alwan A, Mock CN, Nugent R, Watkins D, Adeyi O, et al. Universal health coverage and intersectoral action for health: key messages from Disease Control Priorities, 3rd edition. The Lancet. 2018 2018/03/17/;391(10125):1108-20.

14. Hoch JS, Briggs AH, Willan AR. Something old, something new, something borrowed, something blue: a framework for the marriage of health econometrics and cost-effectiveness analysis. Health Economics. 2002 2002/07/01;11(5):415-30.

15. Frew E, Breheny K. Methods for public health economic evaluation: A Delphi survey of decision makers in English and Welsh local government. Health Economics. 2019 2019/08/01;28(8):1052-63.

16. Mackenbach JP. Re-thinking health inequalities. European Journal of Public Health. 2020 19 June 2020;19 June 2020 (Editorial).

17. Eckard N, Janzon M, Levin L-Å. Use of cost-effectiveness data in priority setting decisions: experiences from the national guidelines for heart diseases in Sweden. International journal of health policy and management. 2014;3(6):323-32. PubMed PMID: 25396208. eng.

18. National Institute for Health and Clinical Excellence. Chapter 7 Incorporating economic evaluation. In: Developing NICE guidelines: the manual. London: National Institute for Health and Clinical Excellence; 2014 [updated 31 October 2018]. Available from: www.nice.org.uk/process/pmg20/chapter/incorporating-economic-evaluation.

19. Sanders GD, Neumann PJ, Basu A, et al. Recommendations for conduct, methodological practices, and reporting of cost-effectiveness analyses: Second panel on cost-effectiveness in health and medicine. JAMA. 2016;316(10):1093-103.

20. Batura N, Pulkki-Brännström A-M, Agarwal P, Bagra A, Haghparast-Bidgoli H, Bozzani F, et al. Collecting and analysing cost data for complex public health trials: reflections on practice. Global Health Action. 2014;7:23257.

For peer review only - http://bmjopen.bmj.com/site/about/guidelines.xhtml

BMJ Open

Protocol for the evaluation of the cost-effectiveness and health equity impact of a school-based tobacco prevention programme in a cluster randomised controlled trial (The TOPAS Study)

Journal:	*BMJ Open*
Manuscript ID	bmjopen-2020-045476.R1
Article Type:	Protocol
Date Submitted by the Author:	24-Mar-2021
Complete List of Authors:	Pulkki-Brännström, Anni-Maria; Umea University, Department of Epidemiology and Global Health<br>Galanti, Maria; Karolinska Institutet, Department of Global Public Health<br>Nilsson, Maria; Umea University, Department of Epidemiology and Global Health
**Primary Subject Heading**:	Health economics
Secondary Subject Heading:	Public health
Keywords:	HEALTH ECONOMICS, PUBLIC HEALTH, PREVENTIVE MEDICINE

SCHOLARONE™
Manuscripts

I, the Submitting Author has the right to grant and does grant on behalf of all authors of the Work (as defined in the below author licence), an exclusive licence and/or a non-exclusive licence for contributions from authors who are: i) UK Crown employees; ii) where BMJ has agreed a CC-BY licence shall apply, and/or iii) in accordance with the terms applicable for US Federal Government officers or employees acting as part of their official duties; on a worldwide, perpetual, irrevocable, royalty-free basis to BMJ Publishing Group Ltd ("BMJ") its licensees and where the relevant Journal is co-owned by BMJ to the co-owners of the Journal, to publish the Work in this journal and any other BMJ products and to exploit all rights, as set out in our licence.

The Submitting Author accepts and understands that any supply made under these terms is made by BMJ to the Submitting Author unless you are acting as an employee on behalf of your employer or a postgraduate student of an affiliated institution which is paying any applicable article publishing charge ("APC") for Open Access articles. Where the Submitting Author wishes to make the Work available on an Open Access basis (and intends to pay the relevant APC), the terms of reuse of such Open Access shall be governed by a Creative Commons licence – details of these licences and which Creative Commons licence will apply to this Work are set out in our licence referred to above.

Other than as permitted in any relevant BMJ Author's Self Archiving Policies, I confirm this Work has not been accepted for publication elsewhere, is not being considered for publication elsewhere and does not duplicate material already published. I confirm all authors consent to publication of this Work and authorise the granting of this licence.

For peer review only

Protocol for the evaluation of the cost-effectiveness and health equity impact of a school-based tobacco prevention programme in a cluster randomised controlled trial (The TOPAS Study)

Anni-Maria Pulkki-Brännström*[1], Maria Rosaria Galanti[2] and Maria Nilsson[1]

[1] Department of Epidemiology and Global Health, Umeå University, Umeå, Sweden

[2] Department of Global Public Health, Karolinska Institutet, Stockholm, Sweden

* Corresponding author: Anni-Maria Pulkki-Brännström, Department of Epidemiology and Global Health, Umeå University, Sweden. anni-maria.pulkki-brannstrom@umu.se

Word count: 2793

For peer review only

ABSTRACT

Introduction

Despite a long-term downward trend in smoking prevalence, tobacco remains the number one risk factor for death and disability in Sweden. Globally, tobacco use generates a substantial economic burden for health systems and is also a major driver of socioeconomic inequalities in health. This paper describes the planned cost-effectiveness and health equity impact evaluation of a multi-component school-based programme to prevent the onset of tobacco use in adolescents.

Methods and analysis

Cost-effectiveness of the multi-component Tobacco-Free Duo programme will be evaluated against the educational component of the same programme only. An incremental cost-effectiveness ratio (ICER) will be calculated in termsof the cost per case prevented using the trial primary outcome and within-trial payer costs. If the ICER is negative, an incremental net benefit ratio will be calculated. Robustness of the results will be assessed through one-way sensitivity analyses. The slope index of inequality will be computed to assess the potential impact of the Tobacco-free Duo programme on education-related inequalities in the onset of smoking and in adult smoking cessation, comparing the two trial arms.

Ethics and dissemination

Ethical approval was obtained from the Regional Ethics Review Board, Umeå (Registration number 2017/255-31). The Public Health Agency of Sweden commissioned the study. The findings will be disseminated internationally within academia and to national and local policy-makers.

Trial registration number ISRCTN 52858080; Pre-results.

STRENGTHS AND LIMITATIONS OF THIS STUDY

- This study uses primary data and an experimental design to address the lack of rigorous economic evaluations of tobacco-prevention programmes.

For peer review only - http://bmjopen.bmj.com/site/about/guidelines.xhtml

- The results will mirror the real costs and cost-effectiveness of the programme, because the intervention is implemented by regular school personnel and integrated into schools' routine tobacco prevention activities.
- The study includes a health equity impact analysis in which the full programme (T-Duo) is compared with structured classroom education only (E).
- Intervention effects are measured on the individual level but resource use is measured on the level of the school.
- The study will not be able to capture the support that the adult "pair" provides to the adolescent in keeping up the "tobacco-free agreement".

For peer review only

For peer review only - http://bmjopen.bmj.com/site/about/guidelines.xhtml

BACKGROUND

Tobacco use is a persistent global public health challenge, and scientific evidence of the effects of tobacco use on mortality and morbidity across the life course is robust. Around 50% of adult smokers die prematurely from tobacco-related disease (1). Smoking is the single most preventable cause of death and one of the leading causes of inequity in health (2). Furthermore, smoking poses large economic costs to individuals, health care systems and the society at large. The World Health Organization has estimated that the economic harm caused by smoking to the world economy is over US$ 500 billion each year (3). The benefits from reduced tobacco use are undisputable, and encompass improved population health, reduced health inequalities, reduced health care costs, and improved economic productivity.

The young person becomes a smoker in a social context (4). Many programmes to prevent young people's tobacco use employ methods based on social influence models. The essence of such models is to change the attitudes, knowledge and behaviour of young people in the context of a social environment. Parents, siblings and friends exert social influence, and the more smokers a young person has in the close context, the more will start smoking (5). Individual and contextual factors influence the process from initiation to regular smoking, and the individual risk of becoming a smoker is impacted by how the different factors relate to and interact with each other.

The need for evidence-based tobacco-prevention programmes targeting young people is large. Programmes implemented outside the health care sector, such as in schools, potentially reach all adolescents, and align well with global calls for multisectoral approaches to promoting health and wellbeing. However, relatively few rigorous evaluations exist. The Swedish agency responsible for recommendations in this area reviewed the literature and found the evidence base to be insufficient, and many evaluation methodologies to be inappropriate, to support recommendations for interventions targeting young people (6). A recent systematic review specifically of the health economic evidence on school-based interventions by Leão et al. (7) found that few evaluations had been published, particularly not recently. There is a

For peer review only - http://bmjopen.bmj.com/site/about/guidelines.xhtml

particular lack of economic evidence from studies that use recently collected primary data (7).

The large socioeconomic inequalities in smoking are well-documented, and parents' low socioeconomic status is a risk factor for adolescent smoking initiation (8). However, a number of studies highlight that socioeconomic inequalities in tobacco use among adolescents are smaller than among adults (9). Furthermore, some studies have found that where prevalence has decreased, socioeconomic inequalities have increased (e.g. (10)). To our knowledge, no evaluation has as yet explicitly evaluated whether a prevention programme has an effect on socioeconomic inequalities in tobacco use among adolescents.

This protocol paper describes the cost-effectiveness and health equity impact evaluation within the TOPAS study. TOPAS is a three-year intervention study that evaluates Tobacco-free Duo, a multi-component school-based programme with public commitment to prevent the onset of tobacco use in adolescents in Sweden. The TOPAS study employs a mixed design approach and consists of a two-arm cluster randomised controlled trial (cRCT) and an observational study. Details of the TOPAS study and the mixed design effectiveness evaluation are described in Galanti et al. (11).

The planned study addresses several of the gaps identified in the evidence base. It is innovative in that an experimental design will be used to evaluate a universal preventive intervention from the perspectives of average health, cost-effectiveness, budget impact, and impact on health inequalities (12). It will provide data to predict system-level intervention costs (7). The current study will be the first to assess the cost-effectiveness of an intervention to prevent adolescent smoking initiation that includes 1) public commitment, and 2) parents or other significant adults (other than school staff). Few previous studies have had a well-defined comparator; in our study the comparator is similar to the one in Tengs et al. (13).

For peer review only - http://bmjopen.bmj.com/site/about/guidelines.xhtml

AIM AND OBJECTIVES

The TOPAS study's health economic evaluation aims to measure the cost-effectiveness and health equity impact of the multi-component Tobacco-free Duo programme (T-Duo). The specific objectives are:

1. To conduct a within-trial analysis of the incremental cost-effectiveness of T-Duo against the structured classroom education component of the same programme (E).
2. To assess whether socioeconomic inequalities in adolescent smoking initiation and in adult smoking cessation differ between T-Duo and E.

For peer review only

METHODS

Study setting and population

The setting for the TOPAS study is secondary schools in nine regions in central Sweden, ranging from small rural communities to major cities. In Sweden, schools are funded through municipality taxes and run by either municipalities or private providers. Healthcare is funded and organised on a regional level. However, municipalities have some public health responsibilities in the area of tobacco prevention, including oversight of national laws and regulations around tobacco, and health promotion in schools.

The main study population consists of adolescents who were in 7th grade in the school year 2018/19 and for whom active consent for data collection has been obtained from a guardian. Secondary study populations are smoking guardians of these adolescents, and all adolescents in 9th grade in the three consecutive school years 2018/19, 2019/20, and 2020/21.

Trial design

In spring 2018, all lower secondary schools with at least two classes in the 7th to 9th grades, and located in 11 regions of central and south Sweden, were invited to participate in the study. An exclusion criteria was whether the school was already planning to adopt the Tobacco-free Duo Programme in the near future. The

For peer review only - http://bmjopen.bmj.com/site/about/guidelines.xhtml

geographical area is large, and includes both urban, semi-urban and rural, sparsely populated areas. School characteristics were collected as part of the baseline data collection to facilitate a comparison of how the schools who self-selected into the study compare with other schools nationally.

Thirty-four schools (clusters) were recruited into the trial and randomised either to the comprehensive Tobacco-free Duo programme (T-Duo) or structured classroom education only (E). The implementation period runs for the three school years from August 2018 to June 2021, when all data collection will be completed. All adolescents who started 7th grade in autumn 2018 are exposed to the intervention for three years; school staff are asked not to expose other year groups to the intervention during the study period. Detailed information about the school recruitment and randomisation processes, and a full description of the interventions is presented elsewhere (11). A brief description of the six components of the Tobacco-free Duo programme, including the structured classroom education component that is common to both trial arms, and the support that the study team provides to schools for implementation, are presented in the following sections.

The multi-component intervention with public commitment

In total 34 schools were randomised to either the Tobacco-free Duo programme (T-Duo; 17 schools), or to the structured classroom education component only (E; 17 schools). Briefly, the overarching aim of the Tobacco-free Duo programme is to prevent tobacco initiation among adolescents using schools as the main arena. T-Duo consists of six components. The first component is the tobacco-free pair, which is a signed agreement between the adolescent and a significant adult. Both parties promise to abstain from tobacco products at least until the adolescent finishes compulsory school. The adolescent is free to choose which significant adult to sign a contract with. Furthermore, the adult is free to choose how best to support the adolescent to keep up the agreement. The programme is introduced by the school staff to students (component 2), and to guardians at a regular parents' meeting (component 3). Adolescents who form a tobacco-free pair and sign a contract receive a membership card (component 4). The card is school-specific and each school decides what benefits or discounts cardholders are entitled to; examples include t-shirts and discounts in the

For peer review only - http://bmjopen.bmj.com/site/about/guidelines.xhtml

school cafeteria. At the end of each school year, all tobacco-free pairs are invited to disclose their tobacco-free status, and adolescents who affirm they have remained tobacco-free enter a prize draw organised by the school (component 5).

Structured classroom education is the sixth component of the Tobacco-free Duo programme, and it is common to both trial arms (T-Duo and E). All schools receive lesson materials for two classroom sessions each term. These consist of age-tailored information and practical exercises about tobacco-related topics, e.g. environmental impact. The materials have been externally commissioned for the study.

Support to school staff for implementation

School staff are responsible for implementation of the interventions in their school. School contact persons receive instructions and materials for each programme component from the study team. However, staff retain a degree of independence regarding timing and exact delivery of the intervention components. Support for implementation is given once a year in the form of a training and networking event. The event is organised by the study team but the training is delivered by external, professional trainers with specific expertise in tobacco prevention in schools. Information about topical tobacco-related issues is given, and upcoming lesson materials for the structured classroom education are introduced. The study team also informs about upcoming data collection. To facilitate data collection and retention of schools in the study, the study team is in regular contact with the schools through monthly newsletters, e-mail and telephone.

Measurement of health outcomes

Health outcome data is collected from adolescents through questionnaires filled in at school, and from guardians through postal questionnaires. Adolescents who change schools are followed up. Adolescents with missing data on the primary outcome will be excluded from the analysis.

The primary outcome in the cRCT is whether the adolescent has *never smoked cigarettes* (negative answer to the question: Did you ever try smoking, even a few puffs?) at the end of 9th grade (smoking onset). The primary outcome, which is self-

For peer review only - http://bmjopen.bmj.com/site/about/guidelines.xhtml

reported, is measured in the main study population among adolescents whose guardians actively consented to data collection at baseline. Adolescents who report at baseline that they have tried smoking, will be excluded from the analysis of the primary outcome. They are however be exposed to and may participate in the intervention on equal terms with others who have never tried smoking.

The cost-effectiveness analysis will use the intention-to-treat results for the trial primary outcome, converted to the number of cases prevented. The average effect of T-Duo compared with E will be expressed as the change in the number of adolescents who have *ever smoked* by the end of 9th grade.

The equity impact analysis will use the trial primary outcome and one of the secondary outcomes, namely parent has quit smoking (no smoking in the past 30 days). This outcome is also self-reported and measured among guardians who were smoking at baseline.

Cost identification, measurement and valuation

Within-trial costs are collected prospectively from a payer perspective throughout the start-up and implementation phases of the cRCT. These costs consist of resource use i) at the school level, and ii) at the level of the programme.

School-level costs are collected primarily through an online data collection tool. A link to the survey is sent by email to the school's contact person approximately once a month during term time throughout the trial start-up and implementation periods. The survey contains questions about completed activities, staff time use, and other direct costs for planning and delivery of the intervention. Questions are included to capture joint costs, such as time used for meetings. Where applicable, data reported by schools is complemented by records held by the study team. Examples of these data include attendance lists for the annual training and networking sessions, and the value of lottery prizes for T-Duo component 5. To value staff time use, average national salaries for the reported staff category will be used.

For peer review only - http://bmjopen.bmj.com/site/about/guidelines.xhtml

Programme costs are collected primarily from study financial accounts. These costs include development of intervention materials and the annual training and networking sessions (e.g. staff, refreshments, venue hire, travel). In order not to underestimate total intervention cost, a small proportion of the time used by the principal investigator and the research assistants who have direct contact with schools is allocated to the intervention. This proportion will be subject to sensitivity analysis. Programme costs will thus cover direct costs and an allocated proportion of study joint costs.

All costs will be valued in Swedish kronor (SEK) and inflated to 2021 values using the consumer price index. An annual 3% discount rate will be applied.

Cost-effectiveness and budget impact analyses

Within-trial school- and programme-level start-up and implementation costs for T-Duo and E will be added up respectively. Total costs will be used to estimate unit costs per school, class, and pupil. A within-trial incremental cost-effectiveness ratio will be calculated by dividing the incremental cost with the number of cases prevented, comparing T-Duo with E. If the resulting ratio is negative, incremental net benefit ratios will be calculated instead (14).

The possibility of modelling budget impact and affordability of national scale-up of Tobacco-free Duo will be explored. For this purpose, the existence of economies of scale at the level of the school will be analysed through the correlation between observed unit costs and the number of pupils or classes in each school.

To systematically explore uncertainty around cost-effectiveness ratios, one-way sensitivity analyses on key assumptions and programme-level parameters will be conducted. Thus robustness of the within-trial results will be analysed with respect to e.g. staff salary levels, the allocation of joint costs, and the inclusion of start-up costs.

Cost per case prevented will be compared with previously published estimates of preventive interventions for adolescents (7). Up-to-date estimates of the societal costs of smoking will be provided to put the results in context. Cost-effectiveness will not

For peer review only - http://bmjopen.bmj.com/site/about/guidelines.xhtml

be compared with threshold values, which are available in Sweden in terms of cost per QALY gained. We will thus not be able to rank T-Duo on the basis of cost-effectiveness against other interventions beyond prevention. We consider that presenting cost per case prevented, together with budget impact and the other study outcomes, will provide sufficient evidence to inform scale-up by national and local decision-makers.

Equity impact analysis

The slope index of inequality will be used to analyse whether the magnitude of socioeconomic inequalities in the health outcomes differs between T-Duo and E. The highest education level attained by the adolescent's guardian(s) will be used to categorise adolescents and guardians from lowest to highest socioeconomic group. The slope index of inequality is a summary measure of absolute inequality that takes into account the relative size of each socioeconomic group (15). The slope index will be adjusted for sex and computed for each health outcome (onset of smoking, adult smoking cessation) for T-Duo and E separately. If there is a statistically significant difference in the index value between T-Duo and E, the hypothesis that T-Duo has no impact on health equity will be rejected.

Patient and public involvement

The intervention under study is a preventive intervention for adolescents and is delivered by regular school staff. School staff are involved in data collection. No patients or members of the public were involved in designing the study.

LIMITATIONS

The TOPAS study will provide rich individual- and school-level data that can be used for further analyses. Of particular relevance may be analyses of costs and consequences beyond the study period; and analyses of relative health inequalities. No decision-analytic modelling is planned as part of this study. However, results for all individual-level health effects (whether positive or negative) among both adolescents and parents from the intention-to-treat analyses in the cRCT will be reported so that

For peer review only - http://bmjopen.bmj.com/site/about/guidelines.xhtml

others may use these results for modelling and subsequently for comparing cost-effectiveness with a wider set of interventions. For example, if the trial finds a significant reduction in the onset of smoking, this result may be used as input into a model of the long-term cost-effectiveness of universal preventive interventions for adolescents, with health outcomes expressed as quality-adjusted life-years. Several models of this type were identified in the review by Leao et al. (7).

Common to many other cluster randomised controlled trials of complex public health interventions, individual-level sensitivity analysis will be neither possible nor conceptually desirable (16). Intervention effects are measured on the individual level, but the intervention is delivered on the level of the school. Thus exposure and resource use are also measured on the school-level. The only exception is that exposure to the core component, the tobacco-free pair, is measured on the individual level (11). However, the resource use associated with this component – what the adult pair does to support the adolescent in keeping up the agreement – will not be captured in this study.

Ethics and dissemination

Ethical approval was obtained from the Regional Ethics Review Board, Umeå, prior to commencement of the study (Registration number 2017/255-31). The results of the study will be disseminated in a scientific article in an international peer-reviewed journal. In addition, results will be disseminated to schools and decision-makers within Sweden through active participation in tobacco prevention –themed events.

Contributorship statement

MRG and MN conceived of and designed the TOPAS study. AMPB and MN conceived the present study. MN acquired the funding. AMPB designed the study, wrote the first draft, and coordinated the write-up of the paper. MN contributed substantially to writing the paper, and together with MRG revised the work critically for important intellectual content. All authors approved of the final version and agree to be accountable for all aspects of the work.

Acknowledgements

For peer review only - http://bmjopen.bmj.com/site/about/guidelines.xhtml

We want to thank the reviewer whose comments helped to clarify the purpose and scope of this protocol paper.

Competing interests

There are no competing interests for any author.

Funding

The study was funded by the Public Health Agency of Sweden, with case reference number 01346-2017 2.3.2.

Data sharing statement

This is a study protocol; data collection is ongoing and no data are as yet available.

REFERENCES

1. Doll R, Peto R, Boreham J, Sutherland I. Mortality in relation to smoking: 50 years' observations on male British doctors. BMJ. 2004;328(7455):1519.
2. World Health Organization. WHO report on the global tobacco epidemic, 2013: enforcing bans on tobacco advertising, promotion and sponsorship. Geneva, Switzerland: World Health Organization, 2013.
3. World Health Organization. Statement of the World Health Organization in relation to the issue of standardized tobacco product packaging. WHO TRIPS Council Meeting; Geneva 28-29 October 2014. Geneva, Switzerland: World Health Organization, 2014.
4. Lakon CM, Hipp JR, Timberlake DS. The social context of adolescent smoking: a systems perspective. American journal of public health. 2010;100(7):1218-28.
5. U.S. Department of Health and Human Services. Preventing Tobacco Use Among Youth and Young Adults: A Report of the Surgeon General. Atlanta, GA: U.S. Department of Health and Human Services, Centers for Disease Control and Prevention, National Center for Chronic Disease Prevention and Health Promotion, Office on Smoking and Health, 2012.
6. SBU. Att förebygga missbruk av alkohol, droger och spel hos barn och unga (Interventions to prevent misuse of alcohol, drugs and gambling addiction in youth). Stockholm: Swedish Agency for Health Technology Assessment and Assessment of Social Services,, 2015 Contract No.: nr 243.
7. Leão T, Kunst AE, Perelman J. Cost-effectiveness of tobacco control policies and programmes targeting adolescents: a systematic review. European Journal of Public Health. 2018;28(1):39-43.
8. Hedman L, Andersson M, Stridsman C, Ronmark E. Evaluation of a tobacco prevention programme among teenagers in Sweden. BMJ Open. 2015;5:e007673. eng.
9. Vallejo-Torres L, Hale D, Morris S, Viner RM. Income-related inequality in health and health-related behaviour: exploring the equalisation hypothesis. Journal of Epidemiology and Community Health. 2014;68(7):615.
10. Sandoval JL, Leão T, Cullati S, Theler J-M, Joost S, Humair J-P, et al. Public smoking ban and socioeconomic inequalities in smoking prevalence and cessation: a cross-

For peer review only - http://bmjopen.bmj.com/site/about/guidelines.xhtml

sectional population-based study in Geneva, Switzerland (1995–2014). Tobacco Control. 2018;27(6):663.
11. Galanti MR, Pulkki-Brännström A-M, Nilsson M. Adult-child contract for universal prevention of tobacco use: Protocol for a mixed-design evaluation of the Swedish school-based program "Tobacco-free Duo" (TOPAS study). JMIR Research Protocols. 2020;9(10):e21100. Pubmed Central PMCID: 7661241.
12. Weatherly H, Drummond M, Claxton K, Cookson R, Ferguson B, Godfrey C, et al. Methods for assessing the cost-effectiveness of public health interventions: Key challenges and recommendations. Health Policy. 2009;93(2-3):85-92.
13. Tengs TO, Osgood ND, Chen LL. The cost-effectiveness of intensive national school-based anti-tobacco education: results from the tobacco policy model. Preventive medicine. 2001;33(6):558-70.
14. Hoch JS, Briggs AH, Willan AR. Something old, something new, something borrowed, something blue: a framework for the marriage of health econometrics and cost-effectiveness analysis. Health Economics. 2002 2002/07/01;11(5):415-30.
15. Mackenbach JP, Kunst AE. Measuring the magnitude of socio-economic inequalities in health: An overview of available measures illustrated with two examples from Europe. Social Science & Medicine. 1997 1997/03/01/;44(6):757-71.
16. Batura N, Pulkki-Brännström A-M, Agarwal P, Bagra A, Haghparast-Bidgoli H, Bozzani F, et al. Collecting and analysing cost data for complex public health trials: reflections on practice. Global Health Action. 2014;7:23257.

For peer review only

For peer review only - http://bmjopen.bmj.com/site/about/guidelines.xhtml

BMJ Open

Protocol for the evaluation of the cost-effectiveness and health equity impact of a school-based tobacco prevention programme in a cluster randomised controlled trial (The TOPAS Study)

Journal:	*BMJ Open*
Manuscript ID	bmjopen-2020-045476.R2
Article Type:	Protocol
Date Submitted by the Author:	23-Jul-2021
Complete List of Authors:	Pulkki-Brännström, Anni-Maria; Umea University, Department of Epidemiology and Global Health<br>Galanti, Maria; Karolinska Institutet, Department of Global Public Health<br>Nilsson, Maria; Umea University, Department of Epidemiology and Global Health
**Primary Subject Heading**:	Health economics
Secondary Subject Heading:	Public health
Keywords:	HEALTH ECONOMICS, PUBLIC HEALTH, PREVENTIVE MEDICINE

SCHOLARONE™
Manuscripts

I, the Submitting Author has the right to grant and does grant on behalf of all authors of the Work (as defined in the below author licence), an exclusive licence and/or a non-exclusive licence for contributions from authors who are: i) UK Crown employees; ii) where BMJ has agreed a CC-BY licence shall apply, and/or iii) in accordance with the terms applicable for US Federal Government officers or employees acting as part of their official duties; on a worldwide, perpetual, irrevocable, royalty-free basis to BMJ Publishing Group Ltd ("BMJ") its licensees and where the relevant Journal is co-owned by BMJ to the co-owners of the Journal, to publish the Work in this journal and any other BMJ products and to exploit all rights, as set out in our licence.

The Submitting Author accepts and understands that any supply made under these terms is made by BMJ to the Submitting Author unless you are acting as an employee on behalf of your employer or a postgraduate student of an affiliated institution which is paying any applicable article publishing charge ("APC") for Open Access articles. Where the Submitting Author wishes to make the Work available on an Open Access basis (and intends to pay the relevant APC), the terms of reuse of such Open Access shall be governed by a Creative Commons licence – details of these licences and which Creative Commons licence will apply to this Work are set out in our licence referred to above.

Other than as permitted in any relevant BMJ Author's Self Archiving Policies, I confirm this Work has not been accepted for publication elsewhere, is not being considered for publication elsewhere and does not duplicate material already published. I confirm all authors consent to publication of this Work and authorise the granting of this licence.

For peer review only

Protocol for the evaluation of the cost-effectiveness and health equity impact of a school-based tobacco prevention programme in a cluster randomised controlled trial (The TOPAS Study)

Anni-Maria Pulkki-Brännström*[1], Maria Rosaria Galanti[2] and Maria Nilsson[1]

[1] Department of Epidemiology and Global Health, Umeå University, Umeå, Sweden

[2] Department of Global Public Health, Karolinska Institutet, Stockholm, Sweden

* Corresponding author: Anni-Maria Pulkki-Brännström, Department of Epidemiology and Global Health, Umeå University, Sweden. anni-maria.pulkki-brannstrom@umu.se

Word count: 2793

For peer review only

For peer review only - http://bmjopen.bmj.com/site/about/guidelines.xhtml

ABSTRACT

Introduction

Despite a long-term downward trend in smoking prevalence, tobacco remains the number one risk factor for death and disability in Sweden. Globally, tobacco use generates a substantial economic burden for health systems and is also a major driver of socioeconomic inequalities in health. This paper describes the planned cost-effectiveness and health equity impact evaluation of a multi-component school-based programme to prevent the onset of tobacco use in adolescents.

Methods and analysis

Cost-effectiveness of the multi-component Tobacco-Free Duo programme will be evaluated against the educational component of the same programme only. An incremental cost-effectiveness ratio (ICER) will be calculated in termsof the cost per case prevented using the trial primary outcome and within-trial payer costs. If the ICER is negative, an incremental net benefit ratio will be calculated. Robustness of the results will be assessed through one-way sensitivity analyses. The slope index of inequality will be computed to assess the potential impact of the Tobacco-free Duo programme on education-related inequalities in the onset of smoking and in adult smoking cessation, comparing the two trial arms.

Ethics and dissemination

Ethical approval was obtained from the Regional Ethics Review Board, Umeå (Registration number 2017/255-31). The Public Health Agency of Sweden commissioned the study. The findings will be disseminated internationally within academia and to national and local policy-makers.

Trial registration number ISRCTN 52858080; Pre-results.

STRENGTHS AND LIMITATIONS OF THIS STUDY

- This study uses primary data and an experimental design to address the lack of rigorous economic evaluations of tobacco-prevention programmes.

For peer review only - http://bmjopen.bmj.com/site/about/guidelines.xhtml

- The results will mirror the real costs and cost-effectiveness of the programme, because the intervention is implemented by regular school personnel and integrated into schools' routine tobacco prevention activities.
- The study includes a health equity impact analysis in which the full programme (T-Duo) is compared with structured classroom education only (E).
- Intervention effects are measured on the individual level but resource use is measured on the level of the school.
- The study will not be able to capture the support that the adult "pair" provides to the adolescent in keeping up the "tobacco-free agreement".

For peer review only

For peer review only - http://bmjopen.bmj.com/site/about/guidelines.xhtml

BACKGROUND

Tobacco use is a persistent global public health challenge, and scientific evidence of the effects of tobacco use on mortality and morbidity across the life course is robust. Around 50% of adult smokers die prematurely from tobacco-related disease (1). Smoking is the single most preventable cause of death and one of the leading causes of inequity in health (2). Furthermore, smoking poses large economic costs to individuals, health care systems and the society at large. The World Health Organization has estimated that the economic harm caused by smoking to the world economy is over US$ 500 billion each year (3). The benefits from reduced tobacco use are undisputable, and encompass improved population health, reduced health inequalities, reduced health care costs, and improved economic productivity.

The young person becomes a smoker in a social context (4). Many programmes to prevent young people's tobacco use employ methods based on social influence models. The essence of such models is to change the attitudes, knowledge and behaviour of young people in the context of a social environment. Parents, siblings and friends exert social influence, and the more smokers a young person has in the close context, the more will start smoking (5). Individual and contextual factors influence the process from initiation to regular smoking, and the individual risk of becoming a smoker is impacted by how the different factors relate to and interact with each other.

The need for evidence-based tobacco-prevention programmes targeting young people is large. Programmes implemented outside the health care sector, such as in schools, potentially reach all adolescents, and align well with global calls for multisectoral approaches to promoting health and wellbeing. However, relatively few rigorous evaluations exist. The Swedish agency responsible for recommendations in this area reviewed the literature and found the evidence base to be insufficient, and many evaluation methodologies to be inappropriate, to support recommendations for interventions targeting young people (6). A recent systematic review specifically of the health economic evidence on school-based interventions by Leão et al. (7) found that few evaluations had been published, particularly not recently. There is a

For peer review only - http://bmjopen.bmj.com/site/about/guidelines.xhtml

particular lack of economic evidence from studies that use recently collected primary data (7).

The large socioeconomic inequalities in smoking are well-documented, and parents' low socioeconomic status is a risk factor for adolescent smoking initiation (8). However, a number of studies highlight that socioeconomic inequalities in tobacco use among adolescents are smaller than among adults (9). Furthermore, some studies have found that where prevalence has decreased, socioeconomic inequalities have increased (e.g. (10)). To our knowledge, no evaluation has as yet explicitly evaluated whether a prevention programme has an effect on socioeconomic inequalities in tobacco use among adolescents.

This protocol paper describes the cost-effectiveness and health equity impact evaluation within the TOPAS study. TOPAS is a three-year intervention study that evaluates Tobacco-free Duo, a multi-component school-based programme with public commitment to prevent the onset of tobacco use in adolescents in Sweden. The TOPAS study employs a mixed design approach and consists of a two-arm cluster randomised controlled trial (cRCT) and an observational study. Details of the TOPAS study and the mixed design effectiveness evaluation are described in Galanti et al. (11).

The planned study addresses several of the gaps identified in the evidence base. It is innovative in that an experimental design will be used to evaluate a universal preventive intervention from the perspectives of average health, cost-effectiveness, budget impact, and impact on health inequalities (12). It will provide data to predict system-level intervention costs (7). The current study will be the first to assess the cost-effectiveness of an intervention to prevent adolescent smoking initiation that includes 1) public commitment, and 2) parents or other significant adults (other than school staff). Few previous studies have had a well-defined comparator; in our study the comparator is similar to the one in Tengs et al. (13).

For peer review only - http://bmjopen.bmj.com/site/about/guidelines.xhtml

AIM AND OBJECTIVES

The TOPAS study's health economic evaluation aims to measure the cost-effectiveness and health equity impact of the multi-component Tobacco-free Duo programme (T-Duo). The specific objectives are:

1. To conduct a within-trial analysis of the incremental cost-effectiveness of T-Duo against the structured classroom education component of the same programme (E).
2. To assess whether socioeconomic inequalities in adolescent smoking initiation and in adult smoking cessation differ between T-Duo and E.

For peer review only

METHODS

Study setting and population

The setting for the TOPAS study is secondary schools in nine regions in central Sweden, ranging from small rural communities to major cities. In Sweden, schools are funded through municipality taxes and run by either municipalities or private providers. Healthcare is funded and organised on a regional level. However, municipalities have some public health responsibilities in the area of tobacco prevention, including oversight of national laws and regulations around tobacco, and health promotion in schools.

The main study population consists of adolescents who were in 7th grade in the school year 2018/19 and for whom active consent for data collection has been obtained from a guardian. Secondary study populations are smoking guardians of these adolescents, and all adolescents in 9th grade in the three consecutive school years 2018/19, 2019/20, and 2020/21.

Trial design

In spring 2018, all lower secondary schools with at least two classes in the 7th to 9th grades, and located in 11 regions of central and south Sweden, were invited to participate in the study. An exclusion criteria was whether the school was already planning to adopt the Tobacco-free Duo Programme in the near future. The

For peer review only - http://bmjopen.bmj.com/site/about/guidelines.xhtml

geographical area is large, and includes both urban, semi-urban and rural, sparsely populated areas. School characteristics were collected as part of the baseline data collection to facilitate a comparison of how the schools who self-selected into the study compare with other schools nationally.

Thirty-four schools (clusters) were recruited into the trial and randomised either to the comprehensive Tobacco-free Duo programme (T-Duo) or structured classroom education only (E). The implementation period runs for the three school years from August 2018 to June 2021, when all data collection will be completed. All adolescents who started 7th grade in autumn 2018 are exposed to the intervention for three years; school staff are asked not to expose other year groups to the intervention during the study period. Detailed information about the school recruitment and randomisation processes, and a full description of the interventions is presented elsewhere (11). A brief description of the six components of the Tobacco-free Duo programme, including the structured classroom education component that is common to both trial arms, and the support that the study team provides to schools for implementation, are presented in the following sections.

The multi-component intervention with public commitment

In total 34 schools were randomised to either the Tobacco-free Duo programme (T-Duo; 17 schools), or to the structured classroom education component only (E; 17 schools). Briefly, the overarching aim of the Tobacco-free Duo programme is to prevent tobacco initiation among adolescents using schools as the main arena. T-Duo consists of six components. The first component is the tobacco-free pair, which is a signed agreement between the adolescent and a significant adult. Both parties promise to abstain from tobacco products at least until the adolescent finishes compulsory school. The adolescent is free to choose which significant adult to sign a contract with. Furthermore, the adult is free to choose how best to support the adolescent to keep up the agreement. The programme is introduced by the school staff to students (component 2), and to guardians at a regular parents' meeting (component 3). Adolescents who form a tobacco-free pair and sign a contract receive a membership card (component 4). The card is school-specific and each school decides what benefits or discounts cardholders are entitled to; examples include t-shirts and discounts in the

For peer review only - http://bmjopen.bmj.com/site/about/guidelines.xhtml

school cafeteria. At the end of each school year, all tobacco-free pairs are invited to disclose their tobacco-free status, and adolescents who affirm they have remained tobacco-free enter a prize draw organised by the school (component 5).

Structured classroom education is the sixth component of the Tobacco-free Duo programme, and it is common to both trial arms (T-Duo and E). All schools receive lesson materials for two classroom sessions each term. These consist of age-tailored information and practical exercises about tobacco-related topics, e.g. environmental impact. The materials have been externally commissioned for the study.

Support to school staff for implementation

School staff are responsible for implementation of the interventions in their school. School contact persons receive instructions and materials for each programme component from the study team. However, staff retain a degree of independence regarding timing and exact delivery of the intervention components. Support for implementation is given once a year in the form of a training and networking event. The event is organised by the study team but the training is delivered by external, professional trainers with specific expertise in tobacco prevention in schools. Information about topical tobacco-related issues is given, and upcoming lesson materials for the structured classroom education are introduced. The study team also informs about upcoming data collection. To facilitate data collection and retention of schools in the study, the study team is in regular contact with the schools through monthly newsletters, e-mail and telephone.

Measurement of health outcomes

Health outcome data is collected from adolescents through questionnaires filled in at school, and from guardians through postal questionnaires. Adolescents who change schools are followed up. Adolescents with missing data on the primary outcome will be excluded from the analysis.

The primary outcome in the cRCT is whether the adolescent has *never smoked cigarettes* (negative answer to the question: Did you ever try smoking, even a few puffs?) at the end of 9th grade (smoking onset). The primary outcome, which is self-

For peer review only - http://bmjopen.bmj.com/site/about/guidelines.xhtml

reported, is measured in the main study population among adolescents whose guardians actively consented to data collection at baseline. Adolescents who report at baseline that they have tried smoking, will be excluded from the analysis of the primary outcome. They are however be exposed to and may participate in the intervention on equal terms with others who have never tried smoking.

The cost-effectiveness analysis will use the intention-to-treat results for the trial primary outcome, converted to the number of cases prevented. The average effect of T-Duo compared with E will be expressed as the change in the number of adolescents who have *ever smoked* by the end of 9th grade.

The equity impact analysis will use the trial primary outcome and one of the secondary outcomes, namely parent has quit smoking (no smoking in the past 30 days). This outcome is also self-reported and measured among guardians who were smoking at baseline.

Cost identification, measurement and valuation

Within-trial costs are collected prospectively from a payer perspective throughout the start-up and implementation phases of the cRCT. These costs consist of resource use i) at the school level, and ii) at the level of the programme.

School-level costs are collected primarily through an online data collection tool. A link to the survey is sent by email to the school's contact person approximately once a month during term time throughout the trial start-up and implementation periods. The survey contains questions about completed activities, staff time use, and other direct costs for planning and delivery of the intervention. Questions are included to capture joint costs, such as time used for meetings. Where applicable, data reported by schools is complemented by records held by the study team. Examples of these data include attendance lists for the annual training and networking sessions, and the value of lottery prizes for T-Duo component 5. To value staff time use, average national salaries for the reported staff category will be used.

For peer review only - http://bmjopen.bmj.com/site/about/guidelines.xhtml

Programme costs are collected primarily from study financial accounts. These costs include development of intervention materials and the annual training and networking sessions (e.g. staff, refreshments, venue hire, travel). In order not to underestimate total intervention cost, a small proportion of the time used by the principal investigator and the research assistants who have direct contact with schools is allocated to the intervention. This proportion will be subject to sensitivity analysis. Programme costs will thus cover direct costs and an allocated proportion of study joint costs.

All costs will be valued in Swedish kronor (SEK) and inflated to 2021 values using the consumer price index. An annual 3% discount rate will be applied.

Cost-effectiveness and budget impact analyses

Within-trial school- and programme-level start-up and implementation costs for T-Duo and E will be added up respectively. Total costs will be used to estimate unit costs per school, class, and pupil. A within-trial incremental cost-effectiveness ratio will be calculated by dividing the incremental cost with the number of cases prevented, comparing T-Duo with E. If the resulting ratio is negative, incremental net benefit ratios will be calculated instead (14).

The possibility of modelling budget impact and affordability of national scale-up of Tobacco-free Duo will be explored. For this purpose, the existence of economies of scale at the level of the school will be analysed through the correlation between observed unit costs and the number of pupils or classes in each school.

To systematically explore uncertainty around cost-effectiveness ratios, one-way sensitivity analyses on key assumptions and programme-level parameters will be conducted. Thus robustness of the within-trial results will be analysed with respect to e.g. staff salary levels, the allocation of joint costs, and the inclusion of start-up costs.

Cost per case prevented will be compared with previously published estimates of preventive interventions for adolescents (7). Up-to-date estimates of the societal costs of smoking will be provided to put the results in context. Cost-effectiveness will not

For peer review only

For peer review only - http://bmjopen.bmj.com/site/about/guidelines.xhtml

be compared with threshold values, which are available in Sweden in terms of cost per QALY gained. We will thus not be able to rank T-Duo on the basis of cost-effectiveness against other interventions beyond prevention. We consider that presenting cost per case prevented, together with budget impact and the other study outcomes, will provide sufficient evidence to inform scale-up by national and local decision-makers.

Equity impact analysis

The slope index of inequality will be used to analyse whether the magnitude of socioeconomic inequalities in the health outcomes differs between T-Duo and E. The highest education level attained by the adolescent's guardian(s) will be used to categorise adolescents and guardians from lowest to highest socioeconomic group. The slope index of inequality is a summary measure of absolute inequality that takes into account the relative size of each socioeconomic group (15). The slope index will be adjusted for sex and computed for each health outcome (onset of smoking, adult smoking cessation) for T-Duo and E separately. If there is a statistically significant difference in the index value between T-Duo and E, the hypothesis that T-Duo has no impact on health equity will be rejected.

Patient and public involvement

The intervention under study is a preventive intervention for adolescents and is delivered by regular school staff. School staff are involved in data collection. No patients or members of the public were involved in designing the study.

LIMITATIONS

The TOPAS study will provide rich individual- and school-level data that can be used for further analyses. Of particular relevance may be analyses of costs and consequences beyond the study period; and analyses of relative health inequalities. No decision-analytic modelling is planned as part of this study. However, results for all individual-level health effects (whether positive or negative) among both adolescents and parents from the intention-to-treat analyses in the cRCT will be reported so that

For peer review only - http://bmjopen.bmj.com/site/about/guidelines.xhtml

others may use these results for modelling and subsequently for comparing cost-effectiveness with a wider set of interventions. For example, if the trial finds a significant reduction in the onset of smoking, this result may be used as input into a model of the long-term cost-effectiveness of universal preventive interventions for adolescents, with health outcomes expressed as quality-adjusted life-years. Several models of this type were identified in the review by Leao et al. (7).

Common to many other cluster randomised controlled trials of complex public health interventions, individual-level sensitivity analysis will be neither possible nor conceptually desirable (16). Intervention effects are measured on the individual level, but the intervention is delivered on the level of the school. Thus exposure and resource use are also measured on the school-level. The only exception is that exposure to the core component, the tobacco-free pair, is measured on the individual level (11). However, the resource use associated with this component – what the adult pair does to support the adolescent in keeping up the agreement – will not be captured in this study.

Ethics and dissemination

Ethical approval was obtained from the Regional Ethics Review Board, Umeå, prior to commencement of the study (Registration number 2017/255-31). The results of the study will be disseminated in a scientific article in an international peer-reviewed journal. In addition, results will be disseminated to schools and decision-makers within Sweden through active participation in tobacco prevention –themed events.

Contributorship statement

MRG and MN conceived of and designed the TOPAS study. AMPB and MN conceived the present study. MN acquired the funding. AMPB designed the study, wrote the first draft, and coordinated the write-up of the paper. MN contributed substantially to writing the paper, and together with MRG revised the work critically for important intellectual content. All authors approved of the final version and agree to be accountable for all aspects of the work.

Acknowledgements

For peer review only - http://bmjopen.bmj.com/site/about/guidelines.xhtml

We want to thank the reviewer whose comments helped to clarify the purpose and scope of this protocol paper.

Competing interests

There are no competing interests for any author.

Funding

The study was funded by the Public Health Agency of Sweden, with case reference number 01346-2017 2.3.2.

For peer review only

Data sharing statement

This is a study protocol; data collection is ongoing and no data are as yet available.

REFERENCES

1. Doll R, Peto R, Boreham J, Sutherland I. Mortality in relation to smoking: 50 years' observations on male British doctors. BMJ. 2004;328(7455):1519.
2. World Health Organization. WHO report on the global tobacco epidemic, 2013: enforcing bans on tobacco advertising, promotion and sponsorship. Geneva, Switzerland: World Health Organization, 2013.
3. World Health Organization. Statement of the World Health Organization in relation to the issue of standardized tobacco product packaging. WHO TRIPS Council Meeting; Geneva 28-29 October 2014. Geneva, Switzerland: World Health Organization, 2014.
4. Lakon CM, Hipp JR, Timberlake DS. The social context of adolescent smoking: a systems perspective. American journal of public health. 2010;100(7):1218-28.
5. U.S. Department of Health and Human Services. Preventing Tobacco Use Among Youth and Young Adults: A Report of the Surgeon General. Atlanta, GA: U.S. Department of Health and Human Services, Centers for Disease Control and Prevention, National Center for Chronic Disease Prevention and Health Promotion, Office on Smoking and Health, 2012.
6. SBU. Att förebygga missbruk av alkohol, droger och spel hos barn och unga (Interventions to prevent misuse of alcohol, drugs and gambling addiction in youth). Stockholm: Swedish Agency for Health Technology Assessment and Assessment of Social Services,, 2015 Contract No.: nr 243.
7. Leão T, Kunst AE, Perelman J. Cost-effectiveness of tobacco control policies and programmes targeting adolescents: a systematic review. European Journal of Public Health. 2018;28(1):39-43.
8. Hedman L, Andersson M, Stridsman C, Ronmark E. Evaluation of a tobacco prevention programme among teenagers in Sweden. BMJ Open. 2015;5:e007673. eng.
9. Vallejo-Torres L, Hale D, Morris S, Viner RM. Income-related inequality in health and health-related behaviour: exploring the equalisation hypothesis. Journal of Epidemiology and Community Health. 2014;68(7):615.
10. Sandoval JL, Leão T, Cullati S, Theler J-M, Joost S, Humair J-P, et al. Public smoking ban and socioeconomic inequalities in smoking prevalence and cessation: a cross-

For peer review only - http://bmjopen.bmj.com/site/about/guidelines.xhtml

sectional population-based study in Geneva, Switzerland (1995–2014). Tobacco Control. 2018;27(6):663.

11. Galanti MR, Pulkki-Brännström A-M, Nilsson M. Adult-child contract for universal prevention of tobacco use: Protocol for a mixed-design evaluation of the Swedish school-based program “Tobacco-free Duo” (TOPAS study). JMIR Research Protocols. 2020;9(10):e21100. Pubmed Central PMCID: 7661241.

12. Weatherly H, Drummond M, Claxton K, Cookson R, Ferguson B, Godfrey C, et al. Methods for assessing the cost-effectiveness of public health interventions: Key challenges and recommendations. Health Policy. 2009;93(2-3):85-92.

13. Tengs TO, Osgood ND, Chen LL. The cost-effectiveness of intensive national school-based anti-tobacco education: results from the tobacco policy model. Preventive medicine. 2001;33(6):558-70.

14. Hoch JS, Briggs AH, Willan AR. Something old, something new, something borrowed, something blue: a framework for the marriage of health econometrics and cost-effectiveness analysis. Health Economics. 2002 2002/07/01;11(5):415-30.

15. Mackenbach JP, Kunst AE. Measuring the magnitude of socio-economic inequalities in health: An overview of available measures illustrated with two examples from Europe. Social Science & Medicine. 1997 1997/03/01/;44(6):757-71.

16. Batura N, Pulkki-Brännström A-M, Agarwal P, Bagra A, Haghparast-Bidgoli H, Bozzani F, et al. Collecting and analysing cost data for complex public health trials: reflections on practice. Global Health Action. 2014;7:23257.

For peer review only

For peer review only - http://bmjopen.bmj.com/site/about/guidelines.xhtml
